# Modèle prédictif de l’échec de la réparation chirurgicale de la fistule obstétricale vésico-vaginale

**DOI:** 10.11604/pamj.2019.34.91.20547

**Published:** 2019-10-16

**Authors:** Joseph Nsambi, Olivier Mukuku, Prosper Kakudji, Jean-Baptiste Kakoma

**Affiliations:** 1 Département de Gynécologie-Obstétrique, Faculté de Médecine, Université de Lubumbashi, Lubumbashi, République Démocratique du Congo; 2 Institut Supérieur des Techniques Médicales, Lubumbashi, République Démocratique du Congo

**Keywords:** Fistule vésico-vaginale, score prédictif, réparation chirurgicale, Vesicovaginal fistula, predictive score, surgical repair

## Abstract

**Introduction:**

Il y a plus de 2 millions de cas de fistule génitale en Afrique subsaharienne et en Asie. Elles surviennent dans les zones où l'accès aux soins à l'accouchement est limité ou de qualité médiocre et où peu d'hôpitaux offrent les interventions chirurgicales correctives nécessaires. L'objectif de cette étude est de développer un score prédictif permettant d'identifier les facteurs influant sur l'échec de la réparation chirurgicale de la fistule obstétricale vésico-vaginale (ERCF) dans la partie Sud-Est de la province du Haut-Katanga.

**Méthodes:**

Il s'agit d'une étude transversale analytique menée sur 384 femmes porteuses d'une fistule obstétricale vésico-vaginale ayant bénéficié d'une prise en charge chirurgicale. Nous avons procédé par une analyse univariée puis multivariée. La discrimination du score était évaluée à l'aide de la courbe Receiving Operating Characteristics (ROC) et du C-index et la calibration du score selon le test d'Hosmer-Lemeshow.

**Résultats:**

La réparation chirurgicale de la fistule obstétricale vésico-vaginale s'était soldée par un échec dans 17,19% des cas (66/384). Après modélisation logistique, quatre critères ressortent comme facteurs prédictifs d'ERCF: la présence d'une fibrose cicatricielle (ORa=15,22; IC95%: 7,34-31,58), la présence de 2 fistules ou plus (ORa=7,41; IC95%: 3,05-17,97), l'abord trans-vésical comme voie d'abord (ORa=4,26; IC95%: 1,92-9,44) et l'atteinte urétrale (ORa=3,93; IC95%: 1,99-7,77). L'aire sous la courbe ROC du score est de 0,8759 avec une sensibilité de 57,58%, une spécificité de 91,82% et une valeur prédictive positive de 91,25%.

## Abstract (en)

**Introduction:**

There are more than 2 million cases of genital fistula in sub-Saharan Africa and in Asia. They occur in people living in areas where the access to medical care during pregnancy and childbirth are limited or of poor quality and where few hospitals can provide adequate surgical repair. The purpose of this study was to develop a score to predict the factors influencing failure in surgical repair of obstetric vesicovaginal fistula (VVF) in the southeastern part of the province of the Upper Katanga.

**Methods:**

We conducted a cross-sectional analytical study of 384 women with vesicovaginal fistula who had undergone surgery. We performed a multivariate and then a univariate analysis. The discrimination of the score was assessed using the ROC curve and the C-index while the calibration of the score using Hosmer-Lemeshow's test.

**Results:**

Surgical repair of obstetric vesicovaginal fistula failed in 17.19% of cases (66/384). After logistic modelling, four criteria emerged as predictors of failure in surgical repair of vesicovaginal fistula: the presence of a fibrotic scar (OR=15.22; CI 95%: 7.34-31.58), the presence of 2 fistulas or more (OR=7.41; CI 95%: 3.05-17.97), transvescical approach (OR=4.26; CI 95%: 1.92-9.44) and urethral involvement (OR=3.93; CI 95%: 1.99-7.77). The area under the ROC curve for the score was 0.8759, with a sensitivity of 57.58%, a specificity of 91.82% and a positive predictive value of 91.25%.

**Conclusion:**

This study demonstrated that the number of fistulas, the presence of fibrotic scar, urethral involvement and transvescical approach are predictors of failure in surgical repair of vesicovaginal fistula.

## Introduction

Une fistule obstétricale (FO) est une communication anormale entre le vagin et les systèmes urologique ou colorectal, causée par un traumatisme obstétrical, qui entraîne une perte incontrôlable d'urine ou de selles [1]. La FO est courante chez les femmes vivant dans des pays à faible revenu, où il se développe généralement à la suite d'un accouchement dystocique et d'un accès insuffisant aux soins prénatals et intrapartum [2]. L'incidence exacte des fistules reste difficile à apprécier car les données statistiques concernant l'ampleur de l'affection sont difficiles à déterminer avec certitude compte tenu de l'absence d'enquêtes épidémiologiques y consacrées. L'Organisation Mondiale de la Santé (OMS) estime que plus de 2 millions de jeunes femmes à travers le monde vivent avec une fistule non traitée et qu'entre 50 000 et 100 000 nouvelles femmes sont touchées chaque année. La grande majorité des cas sont recensés en Afrique subsaharienne et en Asie du sud-est [3,4]. En RDC, les rapports d'Enquête Démographique et de Santé (EDS) de 2007 indiquaient que 0,3% des femmes déclarent avoir déjà éprouvé des symptômes de la fistule [5]. Il n'y a pas dans le monde de classification pronostique universellement reconnue pour les fistules vésico-vaginales; le sujet est difficile car les combinaisons lésionnelles sont multiples. Diverses classifications de la fistule obstétricale ont été élaborées, mais aucune n'a été adoptée au niveau international [6]. Ces classifications se basent sur les différentes caractéristiques anatomopathologiques de la fistule, notamment la distance berge distale fistule-méat urétral, la taille de la fistule, la fibrose cicatricielle, etc [7] et elles ne sont que pédagogiques. Elles ne permettent pas de prédire le résultat de la réparation chirurgicale de la fistule (RCF). Cependant, l'absence d'un système normalisé de terminologie, de classification, de collecte de données et de rapports rend difficile l'évaluation et la comparaison des résultats chirurgicaux. En outre, il existe également des données limitées dans la littérature concernant les caractéristiques de la fistule, de la vessie ou du vagin, ou les techniques de fermeture de la fistule qui prédisent le succès chirurgical [8]. Le taux de réussite après la RCF varie d'un centre à un autre et est déterminé par de nombreux facteurs tels que le site de la fistule, le degré de fibrose cicatricielle, les tentatives de réparation antérieures, la technique de RCF, de même que de l'expertise du chirurgien, de l'équipement et des soins infirmiers post-opératoires entre autres [8-11]. Des taux élevés de réussite après réparation sont rapportés par plusieurs auteurs allant de 72,9 à 93% [12-15]. Cependant, même après la fermeture réussie, 15-20% des cas peuvent continuer à souffrir d´incontinence urinaire et les facteurs prédictifs d´échec comprennent la cicatrisation vaginale, la fistule circonférentielle et les tentatives précédentes de réparation [8,10,16,17]. À l'heure actuelle, dans notre milieu, on ignore encore les différents facteurs contribuant aux résultats de la réparation chirurgicale de la fistule obstétricale vésico-vaginale. Cette étude avait comme objectif d'identifier les facteurs influant sur l'échec de la réparation chirurgicale de la FVV (ERCF) dans la partie sud-est de la province du Haut-Katanga et de développer un score prédictif de l'ERCF.

## Méthodes

**Cadre d'étude:** le Haut-Katanga est depuis 2015 une province de la République Démocratique du Congo à la suite de l'éclatement de la Province du Katanga. Il se situe au sud-est du pays, à la frontière avec la Zambie. Elle comprend six territoires et deux villes à savoir: Kipushi, Mitwaba, Pweto, Sakania, Kasenga, Kambove, la ville de Lubumbashi et celle de Likasi. Ce travail est le fruit de plusieurs campagnes d'opérations chirurgicales gratuites des fistules obstétricales organisées par des organisations non gouvernementales (UNFPA, Médecins du Désert, Médecins sans Frontières Hollande) en collaboration avec le ministère provincial de la Santé Publique du Grand Katanga qui ont voulu donner accès aux soins spécialisés à la population plus précisément à celle habitant dans les zones de santé suivantes : Pweto, Kilwa, Mitwaba, Kasenga, Kashobwe et Lubumbashi. Ces activités ont été réalisées dans les hôpitaux généraux de référence de Kashobwe, Pweto, Mitwaba, Kilwa, Dubié et l'Hôpital Gécamines/Sud de Lubumbashi. Les hôpitaux concernés étaient choisis pour leur accessibilité géographique et pour leur plateau technique ayant rendu possible un grand nombre d'interventions chirurgicales. L'équipe opératoire était composée de deux chirurgiens, un anesthésiste et deux infirmiers. D'autres patientes ont été opérées dans le centre Benicker spécialisée dans la prise en charge des fistules urogénitales. À partir de 2013, cette structure privée a vu le jour dans la ville de Lubumbashi et travaille en collaboration avec une organisation non gouvernementale de droits congolais qui s'appelle Hope Mama Africa. Elle dispense des soins spécialisés dans le domaine de réparation des fistules uro-génitales de manière permanente et permet ainsi de couvrir toute la province du Haut-Katanga. Elle est composée de 4 médecins formés dans la chirurgie vaginale (précisément dans la réparation des fistules) dont un gynécologue-obstétricien, 8 infirmiers et 2 anesthésistes. Cette structure, bien qu'étant privée, est en étroite collaboration avec les zones de santé rurales pour la recherche et la sélection des patientes porteuses de fistules obstétricales.

**Type et période d'étude:** il s'agissait d´une étude transversale analytique examinant les facteurs prédictifs de l'échec de la fermeture suite à la réparation d'une fistule obstétricale vésico-vaginale de 2009 à 2018 dans la province du Haut-Katanga en République démocratique du Congo.

**Population d'étude:** l'étude a porté sur l'effectif total des femmes porteuses de fistule obstétricale vésico-vaginale (survenue après un accouchement par voie vaginale) qui se sont présentées après sensibilisation communautaire dans les villes et villages de la province du Haut-Katanga ainsi que dans leurs environs. La sélection était exhaustive et concernait toute femme porteuse d'une fistule obstétricale vésico-vaginale et remplissant les conditions d'inclusion. Au total, un échantillon de 384 patientes avait été constitué sur base des critères d'inclusion. A été incluse dans l'étude, toute femme porteuse d'une fistule obstétricale vésico-vaginale (survenue lors d'un accouchement par voie basse) reçue en consultation externe ou référée et qui a bénéficié d'une prise en charge chirurgicale au cours de notre période d'étude. Aucun système de classification spécifique de la FVV n'avait été utilisé au cours de la période de l'étude, mais nous avons collecté les caractéristiques anatomopathologiques constituant les différentes classifications existantes. Vu le plateau technique, toutes les femmes porteuses d'une FVV grave (ou transsection) avaient été récusées. Elles étaient au nombre de 22 patientes. Le recueil des données a été fait par l'équipe médicale des structures précitées à l'aide d'un questionnaire préétabli. Ces données ont été recueillies à partir de l'interrogatoire des patientes, des registres des consultations externes, du registre des salles d'opération et des registres d'hospitalisation. Ces documents ont permis d'avoir des renseignements nécessaires sur les femmes depuis leur admission dans le service jusqu'à leur sortie. Toutes les réparations chirurgicales ont été effectuées au bloc opératoire par l'un des 4 médecins de l'équipe de campagne ou du centre Benicker assistés par des médecins généralistes. Après la mise en place d´une anesthésie loco-régionale, les patientes ont été placées en position de Trendelenburg raide, les jambes en lithotomie élevée. Un examen préopératoire a été réalisé dans lequel la fistule, la vessie résiduelle et les caractéristiques vaginales ont été enregistrées.

**Variables étudiées:** les caractéristiques sociodémographiques et gynécologiques des patientes comprenaient l'âge au moment de la réparation chirurgicale (en années) et la parité à la réparation chirurgicale. Les caractéristiques de la fistule comprenaient l'âge de la fistule, le nombre de fistules, la taille de fistule, le type de fistule (vésico-vaginal ou recto-vésico-vaginal), la présence de fibrose cicatricielle (oui ou non), l'état de l'urètre (intact, partiellement ou totalement endommagé) et le nombre de réparations chirurgicales (fistulorraphies) précédentes. On a également enregistré les résultats de la réparation chirurgicale (échec, guérison avec ou sans incontinence urinaire). Les sujets constituant notre population avaient été répartis en deux groupes en fonction du résultat de la RCF. Ce résultat était défini comme suit: 1) échec de la RCF défini comme la non fermeture de la fistule. La fistule n'est pas fermée même si la fuite d'urine a considérablement diminué avec des mictions conservées ou non. 2) succès de la RCF défini comme la fermeture de la fistule; la fistule est totalement fermée, avec ou sans insuffisance sphinctérienne (difficulté de contenir les urines). Il n'y a pas de fuite d'urine à l'endroit où il y avait la fistule.

**Analyse des données:** les différentes données ont été recueillies en utilisant le logiciel Microsoft Excel 2013. Les analyses statistiques ont été faites grâce au logiciel STATA 15. Les données des fistuleuses avec échec de réparation chirurgicale de la fistule ont été comparées à celles dont la réparation chirurgicale s'était soldée par un succès. Ces analyses portaient sur les différentes variables explicatives (variables indépendantes) une à une afin de rechercher une association éventuellement significative avec l'échec de la réparation chirurgicale de la fistule (variable dépendante). La mesure de l'association entre une variable explicative et l'ERCF était faite par le calcul des rapports de côtes (odds ratio) et de leurs intervalles de confiance à 95%. Le test de Chi carré de Pearson a été utilisé pour comparer les proportions observées. La signification statistique a été fixée à p<0,05. Toutes les variables ayant présenté un degré de signification inférieur à 0,2 dans l'analyse unifactorielle ont été intégrées dans une analyse multivariée par régression logistique. Pour la construction du modèle multivarié, on a opté pour la méthode mixte pas à pas (*stepwise selection*) au seuil de p < 0,05. Le modèle logistique a permis ainsi l'analyse de la contribution de chaque variable explicative à l'ERCF en présence des autres variables indépendantes et non la participation des variables explicatives prises isolément. La discrimination du modèle logistique était évaluée par le calcul de l'aire sous la courbe ROC (Receiving Operating Characteristics). L'expression graphique de la discrimination du score se fait par la courbe ROC, qui est le tracé des valeurs de la sensibilité en fonction du complément de la spécificité (1-spécificité). La calibration du score a été faite par le test d'Hosmer-Lemeshow [18]. La discrimination du score est sa capacité à séparer les sujets qui présentent ou non la maladie [19]. On a ensuite déterminé la sensibilité, la spécificité et le pourcentage des cas correctement classés par rapport à la statistique c. L'évaluation de la robustesse des coefficients du modèle a été faite par bootstrap. Un score prédictif du risque a été déduit au terme de l'analyse statistique. En vue de développer un outil de dépistage pour prédire l'ERCF, on a attribué des points à chaque facteur de risque retenu dans le modèle logistique. Pour le rendre simple et utilisable, le score a été estimé en utilisant les valeurs arrondies de ces coefficients [20]. Les probabilités de risque de l'ERFC en fonction des valeurs du score construit ont également été calculées.

**Aspects éthiques:** avant la récolte de données, les autorisations respectives des Médecins Chefs de zone où les patientes étaient recrutées et le Comité d'éthique médicale de l'Université de Lubumbashi avaient été obtenues (N°UNILU/CEM/101/2018). Les données étaient collectées de manière anonyme.

## Résultats

**Figure 1:**
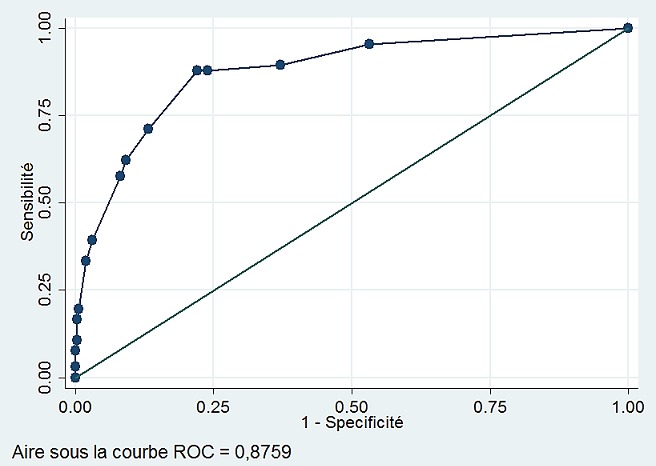
Courbe ROC montrant les performances du score prédictif d´échec de la réparation chirurgicale de fistule obstétricale vésico-vaginale

Au total, 384 patientes porteuses de fistule obstétricale vésico-vaginale ont subi une réparation chirurgicale. Cette dernière s'était soldée par un échec dans 17,19% des cas (66/384). Le Tableau 1 montre qu'il n'y avait pas d'association statistiquement significative entre l'ERCF et l'âge de la patiente, la parité et l'âge de la fistule (p>0,05). Par contre, une association statistiquement significative avait été trouvée entre l'ERCF et l'abord chirurgical, le nombre de fistules, le type de fistules, l'état de l'urètre, la fibrose cicatricielle et la taille de la fistule. L'ERCF était plus importante quand la réparation était faite par abord trans-vésical (ORa=2,15 [1,14-3,98]), en présence de deux fistules ou plus (ORa=9,43 [424-21,03]), quand la fistule a déjà été réparée antérieurement (ORa=2,90 [1,62-5,23]), en présence d'une fistule rectovaginale (ORa=4,03 [1,42-10,90]), quand l'urètre était atteinte partiellement ou totalement (ORa=3,09 [1,7373-5,53]), en présence d'une fibrose cicatricielle (ORa=12,37 [6,47-23,98]) et quand la fistule mesurait 3 cm ou plus (ORa=11,01 [3,14-59,78]). Après modélisation logistique, quatre critères ressortent comme facteurs prédictifs d'échec de la réparation chirurgicale de la FVV (Tableau 2): 1) la présence d'une fibrose cicatricielle (ORa= 15,22 ; IC95% : 7,34-31,58 ; p<0,0001); 2)la présence de 2 fistules ou plus (ORa= 7,41; IC95%: 3,05-17,97; p<0,0001); 3) l'abord trans-vésical comme voie d'abord (ORa= 4,26; IC95% : 1,92-9,44; p<0,0001); 4) l'atteinte urétrale (ORa= 3,93 ; IC95% : 1,99-7,77; p<0,0001). Le score prédictif de l'échec de la réparation chirurgicale de la FVV a été construit à partir du modèle logistique. Chaque facteur de risque a été pondéré par un coefficient de régression représentant le poids de la variable dans le calcul du score, l'ensemble des scores obtenus étant illustré ci-après (Tableau 2). L'aire sous la courbe ROC du score est de 0,8759 (Figure 1). Cette courbe montre une discrimination excellente en ce qui concerne sa capacité de discriminer les patientes qui vont présenter un échec de réparation de celles qui ne vont pas le présenter. La présence de ces quatre critères correspond à un certain nombre de points dont le total est de 7 points. Pour chaque patiente, le score varie de 0 à 7 et au plus il est élevé, au plus le risque d'ERCF est élevé (Tableau 2). Les probabilités de risque d'ERCF en fonction des valeurs du score construit ont été calculées et sont présentées dans le Tableau 3. Un score <4 points définit un groupe de patientes à faible risque d'ERCF; un score entre 4 et 5 points définit un risque modéré d'ERCF et un score >5 points présente un risque élevé d'ERCF. Ainsi pour ce score prédictif d'ERCF, une sensibilité de 57,58% a été obtenue pour une spécificité de 91,82%. La valeur prédictive positive était de 59,38% et la valeur prédictive négative de 91,25%.

**Tableau 1:** Analyse bivariée des facteurs de risque associés à l’échec de la réparation chirurgicale chez les femmes atteintes de fistule obstétricale vésico-vaginale dans la province du Haut-Katanga de 2009 à 2018 en République démocratique du Congo (N=384)

Variable	Echec		Succès		Total	OR brut [95% CI]	p
	n	%	n	%			
**Age lors de la réparation**							
<20 ans	8	11,76	60	88,24	68	1,00	
20-29 ans	34	18,7	148	81,3	182	1,72 [0,75-3,94]	0,2663
≥30 ans	24	17,91	110	82,09	134	1,63 [0,69-3,86]	0,3541
**Parité lors de la réparation**							
1	36	19,25	151	80,75	187	1,00	
≥2	30	15,23	167	84,77	197	1,32 [0,78-2,26]	0,3632
**Âge de la fistule**							
≤1 an	12	19,05	51	80,95	63	1,27 [0,61-2,62]	0,6484
2-10 ans	35	15,63	189	84,38	224	1,00	
>10 ans	19	19,59	78	80,41	97	1,31 [0,71-2,44]	0,4782
**Voie d’abord**							
Trans-vaginale	44	14,57	258	85,43	302	1,00	
Trans-abdominal	22	26,83	60	73,17	82	2,15 [1,14-3,98]	0,0091
**Fistulorraphie antérieure**							
Aucune	35	12,54	244	87,46	279	1,00	
≥1	31	29,52	74	70,48	105	2,90 [1,62-5,23]	<0,0001
**Nombre de fistules**							
1	45	12,93	303	87,07	348	1,00	
≥2	21	58,33	15	41,67	36	9,43 [4,24-21,03]	<0,0001
**Type de fistule**							
Recto-vésico-vaginale	9	42,86	12	57,14	21	4,03 [1,42-10,90]	0,0013
Vésico-vaginale	57	15,70	306	84,30	363	1,00	
**Etat de l’urètre**							
Intact	31	11,74	233	88,26	264	1,00	
Atteinte partiale ou totale	35	29,17	85	70,83	120	3,09 [1,73-5,53]	<0,0001
**Présence de la fibrose cicatricielle**							
Non	19	6,69	265	93,31	284	1,00	
Oui	47	47,00	53	53,00	100	12,37 [6,47-23,98]	<0,0001
**Taille de la fistule**							
<1,5 cm	3	6,12	46	93,88	49	1,00	
1,5-3 cm	28	11,11	224	88,89	252	1,91 [0,55-10,24]	0,4404
>3 cm	35	42,17	48	57,83	83	11,01 [3,14-59,78]	<0,0001

**Tableau 2:** Modèle de régression logistique du risque d’échec de la réparation chirurgicale de fistule obstétricale vésico-vaginale et score de facteurs prédictifs

Variable	OR ajusté	IC à 95%	Coefficient	Score
Fibrose cicatricielle	15,22	7,34-31,58	2,72	3
Nombre de fistule ≥2	7,41	3,05-17,97	2,00	2
Abord trans-vésical	4,26	1,92-9,44	1,45	1
Atteinte urétrale	3,93	1,99-7,77	1,37	1

**Tableau 3:** Probabilité de d’échec de la réparation chirurgicale de fistule obstétricale vésico-vaginale en fonction du score selon le modèle de régression logistique

Score	Probabilité d’ERCF (%)
0	1,82
1	4,25
2	9,64
3	20,39
4	38,07
5	59,62
6	77,99
7	89,49

* Obtenu à partir de la formule: p=1/1 + exp (3,99 – 0,8759 x score)

## Discussion

Dans la présente étude, la fermeture réussie de la fistule obstétricale a été réalisée chez 82,8% des patientes. Dans une étude réalisée en Ouganda, le taux de fermeture réussie était de 77,9% [6]; et dans une autre réalisée en Zambie, ce taux était de 72,9% [14]. Cependant, ces taux de réussite étaient inférieurs à celui rapporté par Hancock (en Ouganda), qui était de 90% [21]. Cela pourrait être dû à la différence clinique de fistules traitées dans ces études. Dans cette étude, le taux de réussite est inférieur aux exigences de l'OMS pour un centre de traitement de la fistule avec plus de 85% de fermeture. Ceci pourrait s'expliquer par le fait que certaines de nos patientes n'ont pas été opérées dans un centre spécialisé de réparation de fistule; par conséquent, la disponibilité des fils de suture et du matériel recommandés ainsi que la qualité des soins postopératoires pouvaient parfois faire défaut. L'étude des facteurs de risque de l'ERCF repose sur la comparaison entre les femmes dont la RCF avait échoué et celles dont la RCF avait réussi. Contrairement aux caractéristiques du patient, des preuves plus solides issues de nos résultats corroborent l'influence négative des caractéristiques de la fistule sur les résultats de la réparation. Dans cette étude, l'ERCF était associée de manière significative à la fibrose cicatricielle, à la présence de 2 fistules ou plus, à l'abord trans-vésical et à l'atteinte urétrale. Cette étude a relevé l'association significative de l'ERCF avec la fibrose circonférentielle cicatricielle qui se crée autour de la fistule. Les études concernant la fibrose cicatricielle ont mis en évidence une association avec les résultats de la RCF, y compris des analyses multivariées démontrant un effet indépendant de la fibrose cicatricielle sur la fermeture [6,8,17,22-25]. Nos résultats fournissent des preuves supplémentaires à l'appui du rôle de la cicatrisation vaginale et de la participation de l'urètre dans la prédiction de l'échec de la fermeture de la fistule. Les femmes présentant une fibrose cicatricielle étaient 15 fois plus susceptibles de ne pas avoir une fermeture de la fistule que celles ne présentant pas de fibrose cicatricielle (ORa= 15,22; IC95%: 7,34-31,58; p<0,0001). Ceci est probablement dû au fait que le tissu cicatriciel a un apport sanguin insuffisant et donc moins susceptible de se refermer. La fibrose rend ainsi la dissection laborieuse. Les fistules avec fibrose cicatricielle sont également difficiles à mobiliser à partir des tissus environnants, ce qui rend une réparation sans tension presque impossible; elles cicatrisent sur les tissus environnants à la suite du processus ischémique ayant conduit à leur formation [6]. Ce constat est aussi confirmé par Zhou *et al*. qui soulignent qu'il est plausible que le tissu cicatriciel puisse entraîner une mobilisation tissulaire limitée pour une réparation sans tension. De plus, l'apport tissulaire et sanguin viable est réduit en présence de fibrose péri-fistulaire modérée ou sévère [26].

Les femmes ayant plus d'une fistule avaient sept fois plus de risque de subir un ERCF que celles ayant une seule fistule (ORa= 7,41; IC95%: 3,05- 17,97; p<0,0001). Il a été confirmé que les fistules multiples (2 ou plus) avaient une relation significative avec la fermeture infructueuse de la FVV [6]. Ceci est probablement dû au fait que la présence de plusieurs FVV rend difficile la mobilisation du tissu local et permet une réparation sans tension en raison du manque de tissu dans la vessie. C'est ainsi que nous pratiquons une suture séparée sans tension de la vessie puis du vagin. Cette technique consiste à dédoubler, après dissection, la vessie et le vagin et à les suturer séparément. Cette séparation du plan vaginal du plan vésical autour de la fistule permet la mobilisation suffisante du plan vésical pour permettre des sutures sans tension après excision des bords scléreux de la fistule. Cette interposition tissulaire permet de combler les espaces morts, de séparer les sutures vaginales et urinaires, d'apporter un tissu bien vascularisé et souple facilitant la cicatrisation et prévenant ainsi un ERCF bien que les résultats de l'étude de Nardos *et al*. n'ont pas trouvé de différence entre les taux d'échec de fistules réparées en un plan et celles réparées en deux plans [8]. La RCF par voie trans-vésicale avait quatre fois plus de chances d'échouer que la réparation par voie trans-vaginale (ORa= 4,26; IC95%: 1,92-9,44; p<0,0001). La RCF par voie trans-vésicale comporte une morbidité accrue due à une perte de sang plus importante, à une durée d'utilisation plus longue, à une longue hospitalisation et à l'entretien de la sonde de Foley. Notre préférence porte sur la voie trans-vaginale car elle offre comme avantages une perte sanguine minime, une durée opératoire plus courte, la possibilité d'interposer un lambeau de Martius ou de muscle droit interne si nécessaire, une faible morbidité postopératoire et une convalescence de courte durée. Trois études ont spécifiquement décrit les résultats des réparations des fistules trans-vaginales versus trans-vésicales, comprenant une cohorte regroupée de plus de 500 patientes [27-29]. Parmi ceux-ci, les taux globaux de fermeture de la fistule étaient de 90,9% et 84,0% respectivement pour les réparations trans-vaginales et trans-vésicales (p<0,05). Les fistules qui surviennent dans les pays en développement sont généralement d'origine obstétricale et sont donc plus susceptibles d'être des FVV basses dans plus de 85% des cas [12]. En conséquence, une proportion beaucoup plus élevée de réparations trans-vaginales sont effectuées dans ce contexte. La voie vaginale expose bien les zones, où seront prélevés les tissus servant aux interpositions entre les sutures vésicales et vaginales. Cette voie comporte un moindre risque lorsque les conditions d'asepsie sont rudimentaires. L'abord trans-vaginal de FVV permet d'obtenir des taux de réussite plus élevés par rapport à l´approche abdominale même si le choix de la voie d'abord est fonction de la complaisance du vagin, du siège de la fistule et des lésions associées. En plus, même pour les fistules haut situées, l'épisiotomie postérieure permet d'abaisser le dôme vaginal et ainsi d'extérioriser la fistule [12]. Les patientes avec atteinte de l'urètre étaient 4 fois plus susceptibles de subir un ERCF (ORa= 3,93 ; IC95%: 1,99-7,77; p<0,0001). Un constat similaire a été observé dans d'autres études [6,8,17,30] où les femmes avec l'atteinte urétrale étaient plus susceptibles d'avoir une défaillance de la réparation chirurgicale de leur fistule que celles sans atteinte urétrale. Cette association pourrait être due au fait que l'urètre est généralement fixé à l'os pubien et donc difficile à mobiliser. Cela pourrait également être dû à la difficulté d'anastomoser un urètre détaché qui est habituellement raccourci par la fistule [6].

La majorité des facteurs de risque associés à l'ERCF décrits dans la littérature sont retrouvés dans la population de l'étude en analyse univariée. Ceci est un élément important de validité externe concernant la définition de la population d'ERCF. Certaines variables connues pour être des facteurs de risque d'ERCF n'ont pas été retrouvées dans cette étude. Il s'agit par exemple de la taille de la fistule, du nombre de fistulorraphies antérieures et de l'association fistule vésico-vaginale et fistule recto-vaginale, qui n'ont pas été retrouvés dans cette population après analyse multivariée. Ceci serait dû probablement au fait que, en les ajustant avec les autres variables, ils paraissent statistiquement moins discriminants et n'ont pas ainsi été retenus dans le modèle prédictif final. L'intérêt de ce score est d'apporter au clinicien un outil lui permettant d'identifier la patiente susceptible de présenter un ERCF (risque individualisé) en vue de mieux la prendre en charge. À notre connaissance, c'est la première étude qui met en relation les facteurs de risque et le résultat de la RCF dans notre environnement en définissant un score. Ce score représente un outil simple qui permettra d'informer, avant chaque RCF, les cliniciens de notre environnement sur la probabilité d'identification des patientes à risque d'un ERCF et pourra ainsi orienter les choix chirurgicaux. Il sera nécessaire de prendre en compte tous ces facteurs lors de la réparation chirurgicale de la fistule afin d'améliorer les résultats, en gardant à l'esprit que la tentative de chirurgie primaire porte toujours les meilleures chances de succès. En effet, ce score ne nécessite que la cotation de 4 éléments. De plus, la bonne spécificité de ce score permet d'écarter la possibilité d'un ERCF. Cependant, pour valider définitivement ce score, la transportabilité dans d'autres populations devra être préalablement testée. La prévention de l'ERCF permettrait aussi celle d'interventions chirurgicales répétées et traumatisantes pour des patientes psychologiquement très affectées, la meilleure chance de succès étant lors de la première tentative de réparation. Tout mettre en œuvre pour réussir du premier coup, car chaque nouvelle intervention engendre une sclérose de plus qui rend plus difficile les tentatives futures. L'identification de la patiente à risque d'ERCF peut aider à prévenir cette dernière dans notre milieu. En effet, les facteurs de risque rapportés dans cette étude doivent attirer l'attention du chirurgien avant toute chirurgie de la fistule. Les décisions de pratique chirurgicale doivent tenir compte des paramètres cliniques de la fistule.

## Conclusion

Les fistules vésico-vaginales obstétricales constituent une grande cause de morbidité maternelle et de ce fait posent un véritable problème de santé publique. Leurs répercussions psycho-sociales sont considérables. En effet, ces femmes, véritables parias à cause de l'odeur des urines insupportable pour leur entourage, sont souvent négligées et abandonnées par leurs conjoints. Cela est d'autant plus dramatique qu'il s'agit de femmes jeunes, en période d'activité génitale. L'approche prédictive de l'ERCF est notre contribution à la réduction de la morbidité des femmes associée à la fistule obstétricale qui représente un véritable calvaire pour les personnes atteintes. Ce modèle logistique a permis d'élaborer un score d'identification du risque de l'ERCF dans un environnement à ressources limitées. Les décisions de pratique thérapeutique pourraient utilement user du score de prédiction de risque d'ERCF proposé en vue d'améliorer la prise en charge des patientes porteuses de fistule obstétricale. L'élimination des fistules obstétricales repose sur la prévention. Cette dernière, pour être efficace, passe par l'instauration de bonnes politiques de santé de la reproduction avec une accessibilité aux services de soins obstétricaux et néonatals de base. Et leur éradication passe par la mise en place d'une véritable politique sanitaire qui s'appuie sur des structures médico-sociales solides correctement réparties sur le territoire national et une formation adéquate des médecins, mais aussi sur des campagnes de sensibilisation des populations à travers les médias, sans surtout oublier leur prévention suivant des pratiques obstétricales basées sur l'évidence.

### État des connaissances actuelles sur le sujet

- La fistule obstétricale est courante chez les femmes vivant dans des pays à faible revenu, où il se développe généralement à la suite d'un accouchement dystocique et d'un accès insuffisant aux soins prénatals et intrapartum;
- L'Organisation Mondiale de la Santé (OMS) estime que plus de 2 millions de jeunes femmes à travers le monde vivent avec une fistule non traitée et qu'entre 50.000 et 100.000 nouvelles femmes sont touchées chaque année;

### Contribution de notre étude à la connaissance

- L'étude proposée est la première étude dans notre ville voire dans notre pays, intégrant une analyse multivariée permettant d'identifier les facteurs de risque d'échec de la réparation chirurgicale de la fistule obstétricale vésico-vaginale dans notre contexte, à Lubumbashi, République Démocratique du Congo;
- Elle est également la première à proposer un outil trouvant son importance dans son utilisation dans le dépistage de femmes porteuses de fistule vésico-vaginale à risque d'échec de réparation chirurgicale dans notre contexte.
